# Transcriptome Analysis of Thermal Parthenogenesis of the Domesticated Silkworm

**DOI:** 10.1371/journal.pone.0135215

**Published:** 2015-08-14

**Authors:** Peigang Liu, Yongqiang Wang, Xin Du, Lusong Yao, Fengbo Li, Zhiqi Meng

**Affiliations:** 1 Sericultural Research Institute, Zhejiang Academy of Agricultural Sciences, Hangzhou, People’s Republic of China; 2 State Key Laboratory Breeding Base for Zhejiang Sustainable Pest and Disease Control, Sericultural Research Institute, Zhejiang Academy of Agricultural Sciences, Hangzhou, People’s Republic of China; Guangzhou Institute of Biomedicine and Health, CHINA

## Abstract

Thermal induction of parthenogenesis (also known as thermal parthenogenesis) in silkworms is an important technique that has been used in artificial insemination, expansion of hybridization, transgenesis and sericultural production; however, the exact mechanisms of this induction remain unclear. This study aimed to investigate the gene expression profile in silkworms undergoing thermal parthenogenesis using RNA-seq analysis. The transcriptome profiles indicated that in non-induced and induced eggs, the numbers of differentially expressed genes (DEGs) for the parthenogenetic line (PL) and amphigenetic line (AL) were 538 and 545, respectively, as determined by fold-change ≥ 2. Gene ontology (GO) analysis showed that DEGs between two lines were mainly involved in reproduction, formation of chorion, female gamete generation and cell development pathways. Upregulation of many chorion genes in AL suggests that the maturation rate of AL eggs was slower than PL eggs. Some DEGs related to reactive oxygen species removal, DNA repair and heat shock response were differentially expressed between the two lines, such as *MPV-17*, *REV1* and *HSP68*. These results supported the view that a large fraction of genes are differentially expressed between PL and AL, which offers a new approach to identifying the molecular mechanism of silkworm thermal parthenogenesis.

## Introduction

Parthenogenesis is the phenomenon production of offspring proceeds without fertilization. As a means of reproduction, parthenogenesis is usually considered an evolutionary dead end, because of the inability to respond genetically to the change of physical and biotic environments. However, parthenogenesis occurs spontaneously in a handful of organisms in nature [1], [2], and is the sole reproductive mode in some organisms [3].

Parthenogenetic reproduction can be either the obligate type or facultative type, which show complex variations between species [4]. Parthenogenesis occurs to some degree in nearly all insect orders through a variety of mechanisms [5], [6]. It has been reported that stick insects (*Phasmida*) reproduce by parthenogenesis, and aphids by a switch between sexual reproduction and parthenogenesis [7], [8]. In addition, parthenogenesis occasionally occurs in the domesticated silkworm (*Bombyx mori*) [9].

*B*. *mori* is a holometabolous lepidopteran insect that has been raised for the purpose of silk production for more than 5,000 years. In most cases, *B*. *mori* females give birth to offspring by mating; however, a few exceptions are reproduced by parthenogenesis without needing a mate [9]. Facultative parthenogenesis in *B*. *mori* was observed as early as the 18th century, and the artificial induction of parthenogenesis was first observed in 1847 by Boursier from female silkworms maintained under sun exposure, and then by Tichomirov in unfertilized eggs treated with sulfuric acid in 1886 [10], [11]. Many experimental treatments have since been proven to be effective in inducing parthenogenesis, including chemicals, oxygenation, electric pulses, mechanical wrapping, centrifugation and cooling [12], [13]. In particular, Astaurov (1940) induced silkworm thermal parthenogenesis by precise spatiotemporal temperature activation (46°C, 18 min) in a water bath of unfertilized eggs [13].

By continuous subculture using an optimized version of Astaurov’s hot-water induction method, the parthenogenetic ability of silkworms can be gradually increased, leading to clones (parthenogenetic lines (PLs)) with high pigmentation rate, high hatching rate, high survival rate and rare abnormal offspring, such that silkworms can be reproduced by parthenogenesis as easily as bisexual breeds reproduce by fertilization [13], [14]. Certain PLs maintained in our laboratory have shown the practical implication of cost reduction of male-only breeding [15]. Some special cross combinations of silkworm (PLs in combination with the sex-linked balanced lethal strains), which produce all-male hybrid progeny, have created a new type of sericulture worldwide. The technique of rearing only male silkworms in rural areas and rearing more female silkworms in egg-producing stations is very important to improve the yield and quality of cocoon silk, and to reduce the production costs of male silkworm hybrid eggs.

Silkworm parthenogenesis research has mainly focused on the induction method and construction of PLs, with few studies on the mechanism [16]. Astaurov’s hot-water induction method is very effective to induce silkworm parthenogenesis; however, its molecular mechanism remains unclear. In silkworm thermal parthenogenesis, all parthenogenetic progeny are females with their maternal genotype being repeated or cloned, in theory [13]. Although parthenogenetic offspring copy the maternal genotype during thermal parthenogenetic induction, variations in parthenogenetic ability (pigmentation rate, hatching rate, survival rate and abnormal rate) occur in the inductive process and the mechanism is poorly understood.

The parthenogenetic ability of silkworms can increase after long-term selection. It is hypothesized that the selected eggs’ transcriptomes would differ from those of the non-selected eggs. Characterization of the general differences between stable PL and its original parent the amphigenetic line (AL) could help to explain the differences in parthenogenetic ability between them. To this end, we employed RNA-seq to characterize the transcriptome differences between PL and AL before and after thermal induction. We observed that a number of transcripts were differentially regulated between the two lines at each time interval. The potential effects of these differences in egg gene expression on the differences in parthenogenetic ability are discussed. These findings are very important to understand the intracellular signaling mechanisms of silkworm thermal parthenogenesis.

## Methods

### Egg sampling and hot-water induction

The silkworm strains, Wu 14 (PL) and 54A (AL), maintained in the Sericultural Research Institute of Zhejiang Academy of Agricultural Sciences, were used in this study. 54A is an important Japanese AL that reproduces by mating from generation to generation. Wu 14 is a stable PL that reproduces by parthenogenetic induction and was obtained from female moths of 54A through several generations of selection by the hot-water induction method (46°C, 18 min) of Astaurov [13]. Before RNA-seq was employed, Wu 14 had experienced 23 generations of hot-water inductive selection. The insects were reared at 25°C and 70–80% relative humidity (RH). Five hundred larvae were reared in one feeding-tray and fed with the same weight of mulberry leaves. Eggs representing distinct stages of development were collected from at least 40 female individuals and dissected out.

Eleven hours after eclosion of the female moths, the non-induced eggs were obtained by dissecting the female moths and rinsed using room temperature water. After drying, one-third of the collected eggs were immersed quickly in liquid nitrogen and stored at −70°C; the remainder were soaked in a water bath at 46°C for 18 min and rapidly cooled in a water bath at 25°C for 3 min. The induced eggs were air dried and divided into two groups: one group was immersed quickly in liquid nitrogen; and the other was stored at 16°C under 80% RH for 3 d for the statistical analysis of parthenogenetic ability.

Four egg samples were prepared for RNA-seq analysis: non-induced AL eggs (ALUI_eggs), non-induced PL eggs (PLUI_eggs), hot-water induced AL eggs (ALHI_eggs) and hot-water induced PL eggs (PLHI_eggs).

### Library construction and high-throughput sequencing

Silkworm eggs (0.2 g) were collected from each sample for RNA extraction. Total RNA extraction was performed using the TRIzol reagent, following the manufacturer’s instructions (Ambion, Foster City, CA, USA). The total RNA concentration was determined using a Qubit RNA Assay Kit in Qubit 2.0 Flurometer (Life Technologies, Carlsbad, CA, USA) and the quality of the RNA samples was assessed by agarose gel electrophoresis.

RNA library construction was performed by Novogene Bioinformatics Technology Co., Ltd, Beijing, China (http://www.novogene.cn/). Before the library construction, the integrity of the RNA samples was confirmed using an RNA Nano 6000 Assay Kit in the Agilent Bioanalyzer 2100 system (Agilent Technologies, Santa Clara, CA, USA). The mRNA was purified from about 3 μg of total RNAs using poly-T oligo-attached magnetic beads. Fragmentation was carried out using divalent cations at 94°C for 5 min in NEBNext First Strand Synthesis Reaction Buffer (5×). First strand cDNA was synthesized using random hexamer primers and M-MuLV Reverse Transcriptase (RNase H-). Second strand cDNA synthesis was subsequently performed using DNA polymerase I in RNase H. Remaining overhangs were converted into blunt ends via exonuclease/polymerase activities. After adenylation of the 3 ends of the DNA fragments, a NEBNext Adaptor with a hairpin loop structure was ligated to prepare for hybridization. To select cDNA fragments of the preferred 150–200 bp in length, the library fragments were purified using the AMPure XP system (Beckman Coulter, Beverly, CA, USA). Then, 3 μl USER Enzyme (NEB, Ipswich, MA, USA) was used with size-selected, adaptor-ligated cDNA at 37°C for 15 min, followed by 5 min at 95°C before polymerase chain reaction (PCR). PCR was performed with Phusion High-Fidelity DNA polymerase, universal PCR primers and the Index (X) Primer. Finally, PCR products were purified (AMPure XP system) and library quality was assessed using the Agilent Bioanalyzer 2100 system.

### Reads mapping to the reference genome

The raw reads in the fastq format were first processed using in-house perl scripts. In this step, clean reads were obtained from the raw reads by removing reads containing adapters, reads containing poly-N and low-quality reads (quality limit 0.05). The clean, high-quality reads were used for downstream analyses. At the same time, the Q20, Q30 and GC contents of the clean data were calculated.

The reference genome and gene model annotation files of *B*. *mori* were downloaded from the genome website (http://www.silkdb.org/silkdb) [17]. An index of the reference genome was built using Bowtie (version 2.0.6) and paired-end clean reads were aligned to the reference genome using TopHat (version 2.0.9). The transcriptome coverage was deduced using the transcriptome data in this study (4.34–4.70 Gb) divided by the standard silkworm genome data (432 Mb) of the International Silkworm Genome Consortium.

### Bioinformatic analysis of RNA-seq data

The reads number mapped to each gene was counted using HTSeq (version 0.5.4p3). The reproducing kernel particle method (RKPM) value of each gene was calculated based on the length of the gene and read count mapped to this gene [18].

Differential expression analysis of the two lines was performed using the DESeq R package (1.12.0). The P-values were adjusted using the Benjamini & Hochberg method [19]. A corrected P-value of 0.005 and a log2 (fold-change) of 1 were set as the thresholds for significantly differential expression.

### Validation of RNA-Seq by quantitative real-time reverse transcription polymerase chain reaction (qRT-PCR)

To validate DEGs in the libraries, seven DEGs were selected for qRT-PCR confirmation. The primer sequences and related information are shown in S1 Table.

According to the SYBR Premix Ex Taq Kit (TaKaRa, Shiga Pref, Japan) protocol, the reactions were run on an Opticon lightcycler (BioRad, Hercules, CA, USA) using a 20-μL reaction system. The reaction conditions were: 95°C for 5 s; followed by 45 cycles at 60°C for 10 s and 72°C for 10 s. All samples were performed in triplicate. The cycle threshold (Ct) values obtained from 18S rRNA (a housekeeping gene of silkworm) amplification in the same plate were used to normalize the relative expression levels. The data of relative expression levels were analyzed and normalized relative to 18S rRNA transcript levels using the Opticon Monitor analysis software (MJ Research, Waltham, MA, USA). The relative gene expression of four samples was calculated using the 22DDct method [20].

### GO and KEGG pathway enrichment analyses

Gene ontology (GO) enrichment analysis of DEGs was implemented by the GOseq R package (version 1.10.0), in which gene length bias was corrected. GO terms with corrected P-values less than 0.05 were considered significantly enriched by DEGs. The KOBAS software, available from http://kobas.cbi.pku.edu.cn/home.do, was used to test the statistical enrichment of DEGs in Kyoto Encyclopedia of Genes and Genomes (KEGG) pathways analysis. KEGG pathways with a corrected P-value less than 0.05 were considered significantly enriched by DEGs.

## Results

### Differential phenotypes of PL and AL

**Fig 1:**
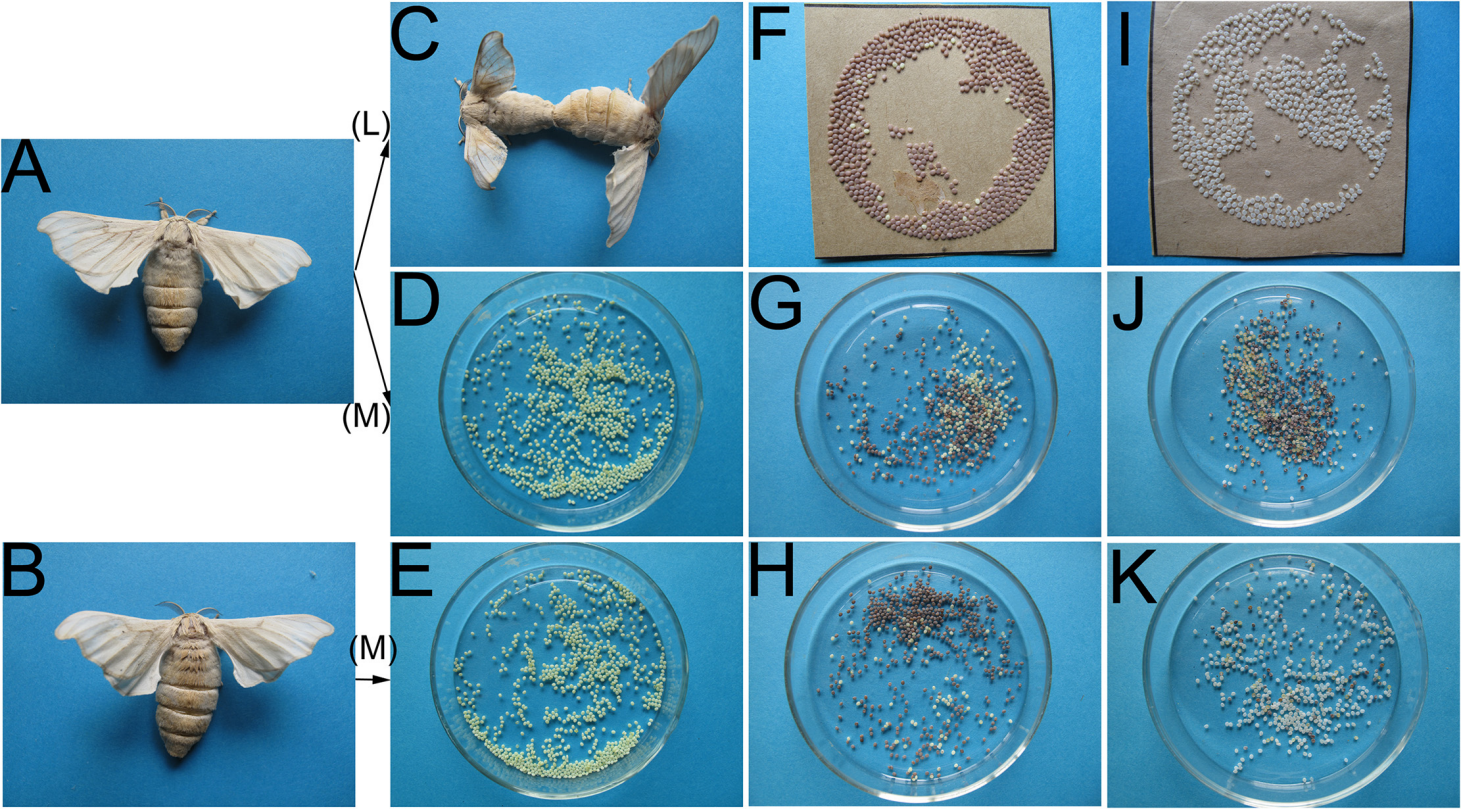
Comparison of parthenogenetic ability and fertilization between PL and AL. (A) and (B) The virgin female moths of 54A and wu 14, respectively. (C) Selfing by mating of 54A. (D) and (E) Non-thermally induced eggs of 54A and wu 14 dissected from the virgin female moths, respectively. (F), (G) and (H) Pigmentation rate of 54A (selfing), 54A (parthenogenetic induction) and wu 14 (parthenogenetic induction), respectively. (I), (J) and (K) Hatching rate of 54A (selfing), 54A (parthenogenetic induction) and wu 14 (parthenogenetic induction), respectively. (L) Selfing through mating. (M) Parthenogenetic induction.

PL was obtained from female moths of AL through several generations of selection by the thermal inductive method. Our previous studies demonstrated that PL was superior to AL in the parthenogenetic ability after more than 20 generations of selection, mainly manifested in four aspects: the pigmentation rate, the hatching rate, the survival rate and abnormal rate of larvae [21]. Unfertilized eggs of PL and AL subjected to the same thermal parthenogenetic progress displayed differential parthenogenetic abilities. In terms of reproductive ability and vitality, PL was similar to AL (selfing) in which the female gives birth to offspring by mating with a male (Fig 1). In terms of the pigmentation rate of thermally induced eggs, PL was higher than AL, and there were significant differences between AL individuals. In contrast, there were no significant differences between PL individuals. Most pigmented eggs from the AL were shriveled and died during the period of silkworm eggs protection; therefore, the hatching rate of AL was significantly lower than that of PL; indeed, some eggs of AL moths hatched no larva. Artificial extrusion and milling harm eggs, which reduce the inducibility; therefore, in thermally induced eggs, the pigmentation rate and hatching rate of PL were slightly lower than in the fertilized eggs of AL (selfing). The results of parthenogenetic ability obtained in October 2013 are shown in Table 1. The differences in the parthenogenetic abilities of the two lines are shown in Fig 1.

**Table 1:** Parthenogenetic ability between PL and AL.

Variety	Pigmentation rate (%)^a^	Hatching rate (%)^b^	Survival rate (%)^c^	Abnormal rate (%)^d^
54A (AL, selfing)	99.83	98.48	89.20	0
54A (AL, parthenogenetic induction)	60.51	4.37	11.20	18.79
wu14 (PL, parthenogenetic induction)	89.46	81.78	78.50	0

Notes: The data of the pigmentation rate and hatching rate are the average value of 20 sets of eggs (one set of eggs laid by one moth). The survival rate of parthenogenetic offspring was obtained from 500 individuals, and the abnormal rate of parthenogenetic offspring was obtained from 300 individuals (AL of parthenogenetic induction only have 297 individuals). The pigmentation rate data were obtained in October 2013 and the hatching rate and abnormal rate data were obtained in May 2014, respectively.

^a^ Pigmentation rate is the ratio of the number of pigmented eggs to the total number of eggs treated.

^b^ Hatching rate is the ratio of the number of eggs that hatched into silkworms to the total number of eggs treated.

^c^ Survival rate is the ratio of developed complete silkworms (egg to moth) to the total number of parthenogenetic offspring.

^d^ Abnormal rate is the ratio of abnormal individuals to the total number of parthenogenetic offspring; the number of abnormal individuals was obtained on the 3^rd^ day of the 5^th^ instar of the larvae.

Only 11.2% (56 moths obtained from 500 newly-hatched silkworms) of the parthenogenetic offspring of AL developed completely from egg to moth, much less than the parthenogenetic offspring of PL and amphigenetic offspring of AL. We also found that the parthenogenetic offspring of AL contained 18.8% (56 abnormal individuals to 298 individuals of 3^rd^ day of the 5^th^ instar) abnormal individuals, while no abnormal individuals were found in the offspring of PL. Results of survival rate and abnormal rate are listed in Table 1.

### Mapping of RNA-seq reads to silkworm genome

Using RNA-seq, it is possible to characterize the transcriptomic landscapes of PL and AL. To accomplish this, two rounds of linear amplification of mRNA were carried out to obtain sufficient RNA input of individual eggs for analysis. Amplified RNAs, all with the same sire, from 0.2 g eggs (approximately 400 eggs) of PL and AL were pooled, multiplexed, and sequenced on the HiSeq2000 (Illumina, San Diego, CA, USA). High-throughput sequencing generated 45.36–53.26 million (M) raw reads for each sample. The total length of the clean reads was 4.34–4.70 gigabases (Gb) after quality filtering, representing more than 10-fold coverage of the *B*. *mori* genome and more than 130-fold coverage of the annotated transcriptome.

After quality filtering, all short reads were mapped onto the *B*. *mori* genome using TopHat [17]. The ratio of reads that could be uniquely aligned to the genome was 76.41%–77.94%, in which approximately 55% of the reads were mapped to known exons and 22% were located in predicted intergenic or intronic regions (Table 2).

**Table 2:** Statistics for filtering and mapping reads.

Sample name	ALUI_eggs	ALHI_eggs	PLUI_eggs	PLHI_eggs
Raw reads	45642086	45367550	48829078	53267444
Q20	96.73	96.65	96.96	96.95
Q30	90.73	90.48	91.07	91.08
GC Content(%)	44.27	44.94	43.72	43.73
Clean reads	43667962	43490662	47090892	50396006
Total mapped	34595613 (79.22%)	34905084 (80.26%)	37608858 (79.86%)	39878143 (79.13%)
Multiple mapped	1228265 (2.81%)	1008424 (2.32%)	1034802 (2.2%)	809244 (1.61%)
Uniquely mapped	33367348 (76.41%)	33896660 (77.94%)	36574056 (77.67%)	39068899 (77.52%)
Non-splice reads	24256073 (55.55%)	23843258 (54.82%)	26266881 (55.78%)	27936302 (55.43%)
Splice reads	9111275 (20.86%)	10053402 (23.12%)	10307175 (21.89%)	11132597 (22.09%)

### Analysis of DEGs

**Fig 2:**
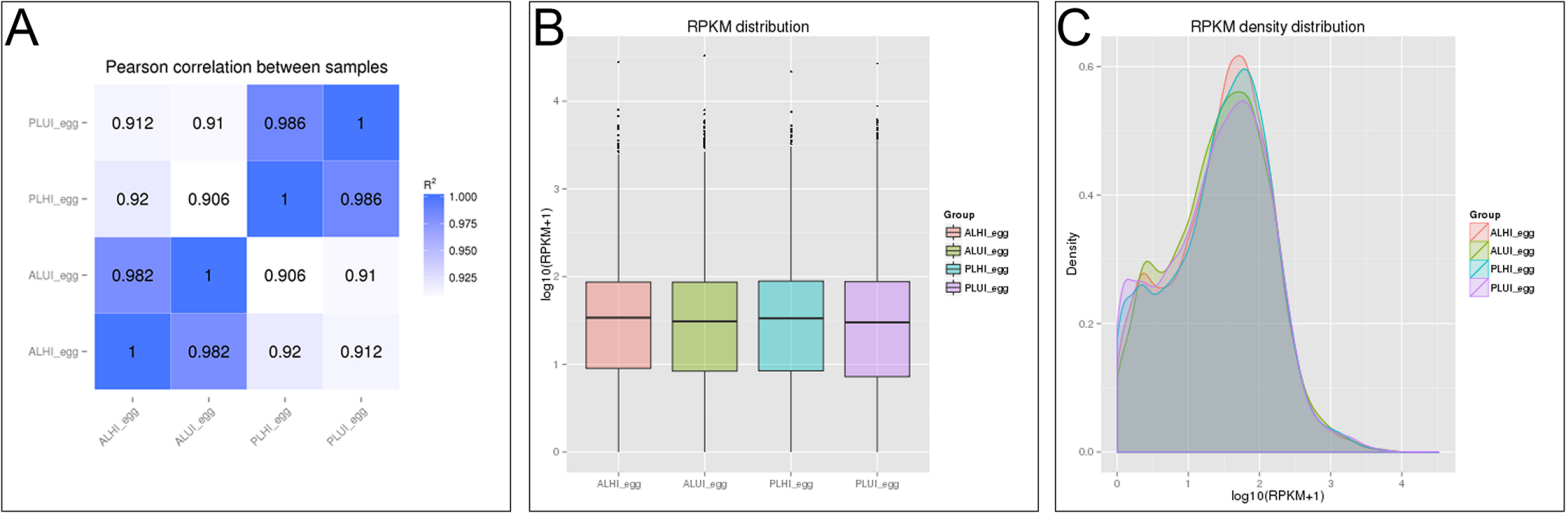
Bioinformatic analyses of RNA-seq data. (A) Pearson correlation between four sets of egg samples. (B) Reproducing kernel particle method (RKPM) distribution of four sets of egg samples. (C) RPKM density distribution of four sets of egg samples.

Before DEGs analysis, Pearson correlation between samples was determined by the RNA-seq correlativity analysis, and the results are shown in Fig 2A. The expression similarity between samples was very close and the sample selection in this study was reasonable (R^2^ > 0.8). Subsequently, the mapping data generated by TopHat, transcript assembly, and differential expression were analyzed using the Cufflinks software. The abundance of gene transcripts was expressed as reads per kilobase of transcript per million fragments mapped (RPKM) [18]. The results of RPKM distribution and RPKM density distribution of the four samples are shown in Fig 2B and 2C, respectively.

**Fig 3:**
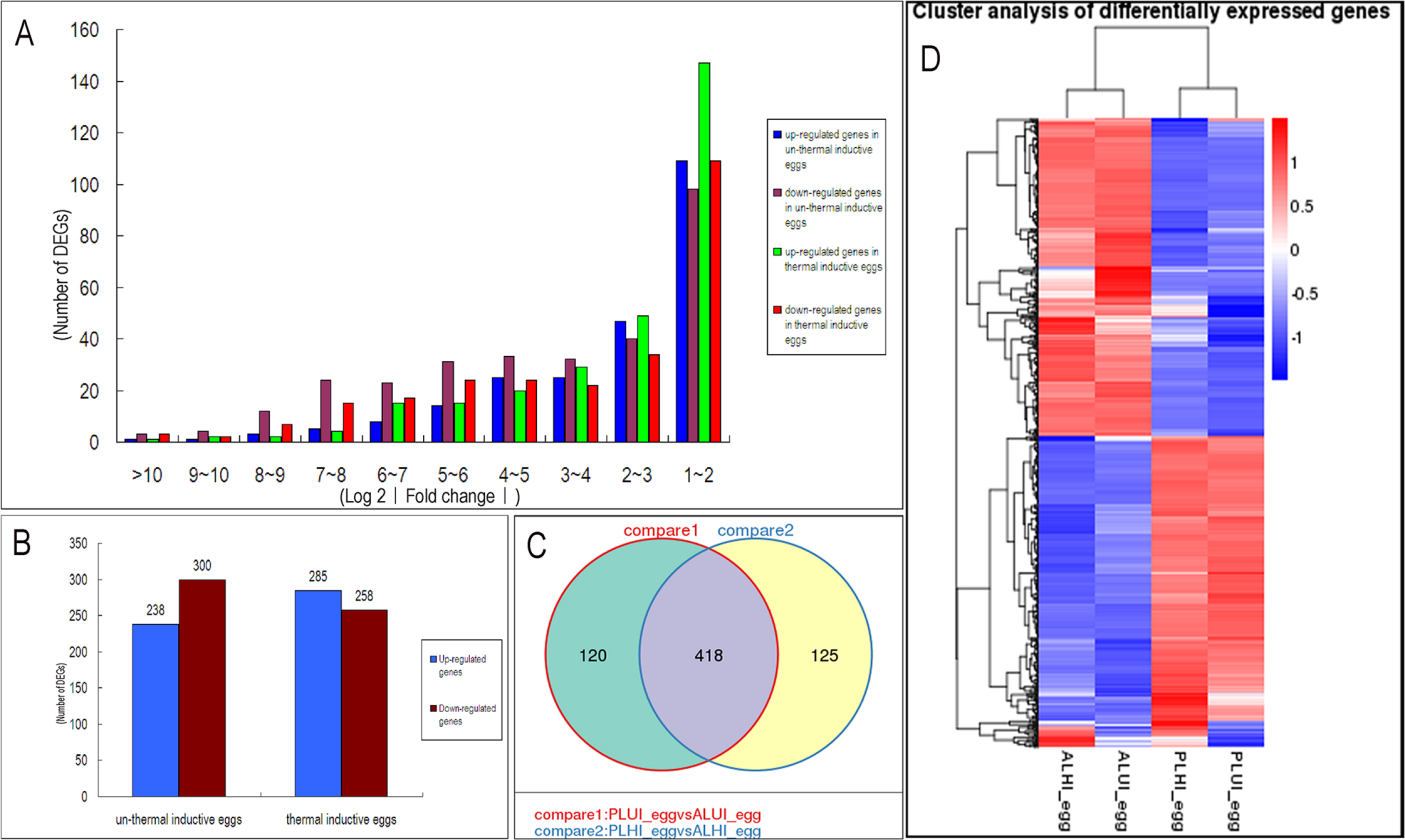
Bioinformatic analysis of DEGs. (A) The fold-change distribution of DEGs. (B) Number of up-regulated and down-regulated genes in non-induced and thermally induced eggs. (C) Venn diagrams showing the number of DEGs between the two lines before and after thermal induction. (D) Cluster analysis of DEGs.

Genes between AL and PL with fold-change ≥ 2, P-value > 0.05 and q-value < 0.05 were considered to be differentially expressed. The number of DEGs is summarized in Table 3 and the fold-change distribution of DEGs is shown in Fig 3A. Setting AL as the comparison, in non-thermally induced eggs, fewer genes were upregulated in PL than were downregulated, while small differences were observed between the upregulated and downregulated genes in thermally induced PL eggs (Fig 3B).

**Table 3:** Statistics of genes regulated between two lines.

Classification	DEGs of Compare 1	DEGs only belong to Compare 1	DEGs belong to Compare 1 and Compare 2	DEGs only belong to Compare 2	DEGs of Compare 2
**Upregulated genes**	238	27	211	74	285
**Downregulated genes**	300	93	207	51	258

Note: Compare 1: PLUI_eggs *vs*. ALUI_eggs, Compare 2: PLHI_eggs *vs*. ALHI_eggs.

The gene expression data for the non-thermally induced eggs showed that a total of 538 DEGs were identical between PL and AL (S2 Table), of which 238 DEGs were upregulated and 300 DEGs were downregulated in PL. Before thermal induction, genes such as *clavesin-1* (*CLVS1*), *enkurin* (*ENKUR*), *putative alpha-L-fucosidase* (*FUCO*) and *metabotropic glutamate receptor 7* (*GRM7*) were highly expressed in PL. In AL, regulated genes, including some chorion family genes, such as *chorion class A protein L12* (*CHA2*), *chorion class CB protein M5H4* (*CHCB1*) and *chorion class CA protein ERA*.*1* (*CHCA1*), were highly expressed.

In thermally induced eggs, the number of up and downregulated genes of PL were 285 and 258, respectively. The statistics of DEGs in thermally induced eggs are shown in S3 Table. After thermal induction, *CLVS1*, *ENKUR*, *FUCO* and *cyclic nucleotide-gated cation channel beta-1* (*CNGB1*) were highly expressed in PL, while genes such as *chorion class B protein L12* (*CHB2*), *CHA2*, *Bardet-Biedl syndrome 5 protein homolog* (*BBS5*), *chorion class A protein L11* (*CHA1*) and *myrosinase 1* (*MYRO1*) were highly expressed in AL.

Venn diagram analysis of the DEGs between the PL and AL lines in non-induced and induced eggs revealed that 418 DEGs were present in both types of eggs, in which 120 DEGs displayed expression differences only in non-induced eggs and 125 gene expression differences induced eggs. The results of Venn diagram analysis are displayed in Fig 3C and the statistics of DEGs classification are listed in S4 Table.

The DEGs cluster analysis (Fig 3D) showed that DEGs could be classified into three groups, comprising two large groups and a small one. Interestingly, in the two large groups, one group was mainly upregulated in AL and downregulated in PL, and the other group displayed the opposite expression pattern. The small group contained DEGs that were upregulated in non-induced eggs and downregulated in induced eggs in the two lines.

### Validation by qRT-PCR

To validate the expression profiles from the RNA-seq analysis, the relative mRNA levels of seven genes that displayed significant differences between the two lines were analyzed using qRT-PCR. The fold-changes of the results for RNA-seq and qRT-PCR are compared in Table 4. The expression trends of most of the genes in RNA-seq were consistent with those from qRT-PCR. The fold-changes were different between qRT-PCR and RNA-seq, which could be attributed to the different probes used for qRT-PCR and RNA-seq.

**Table 4:** Comparisons between RNA-seq data and qRT-PCR results.

Gene name	Fold change			
	PLUI_eggvsALUI_egg		PLHI_eggvsALHI_egg	
	qRT-PCR	RNA-seq	qRT-PCR	RNA-seq
*60S ribosomal protein L29*	1.89	36.00	6.44	56.86
*Heat shock protein 68*	1.16	-6.15	-4.01	-8.11
*Protein Mpv17*	1.67	2.87	27.67	3.76
*Cysteine synthase*	-0.18	17.14	21.60	19.03
*Ribokinase*	-2.11	28.64	8.51	<2
*Purine nucleoside phosphorylase*	1.24	25.46	189.49	96.34
*Chorion class B protein L11*	-1.62	-163.14	-3.41	-328.56

### GO enrichment analysis of genes

DEGs between PL and AL in non-induced and induced eggs were analyzed and categorized functionally based on three GO categories at P-values ≤ 0.05 using Blast2GO. The results of the GO term analysis in non-induced and induced eggs are shown in S5 Table and S6 Table, respectively.

**Fig 4:**
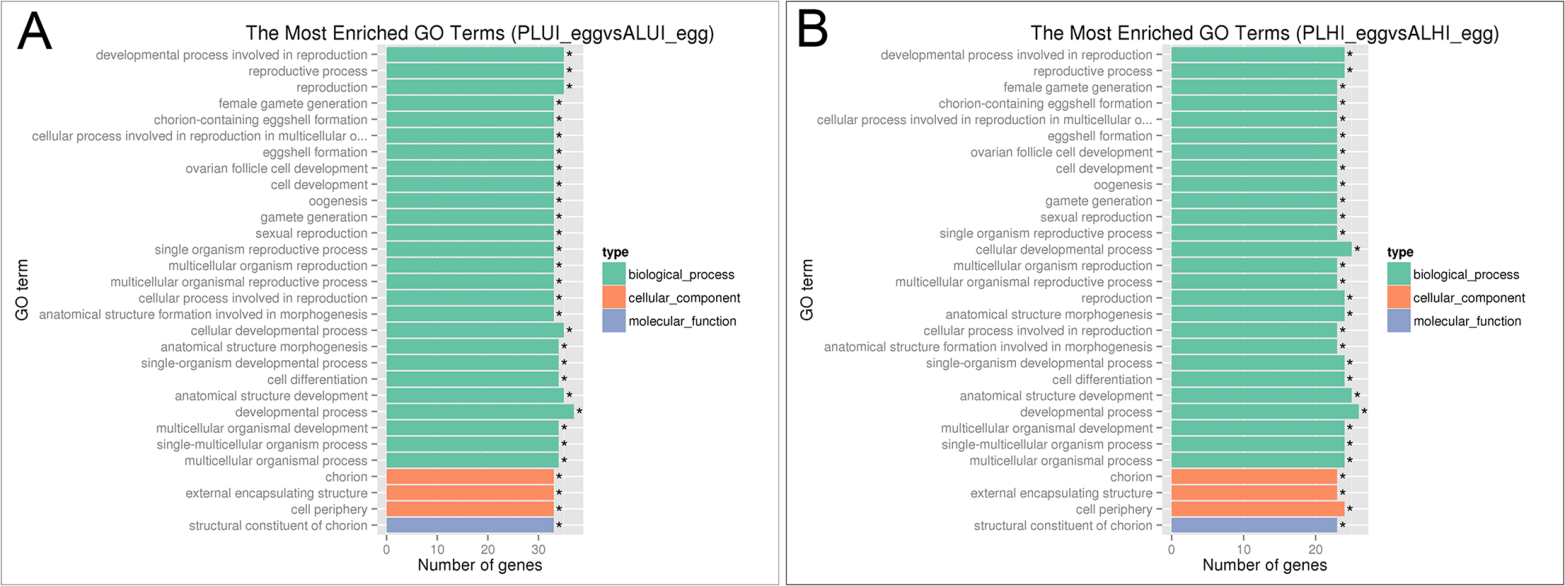
GO bar chart of DEGs between PL and AL. (A) The most enriched GO terms for DEGs between two the lines in non-thermally induced eggs. (B) The most enriched GO terms for DEGs between two lines in thermally induced eggs.

**Fig 5:**
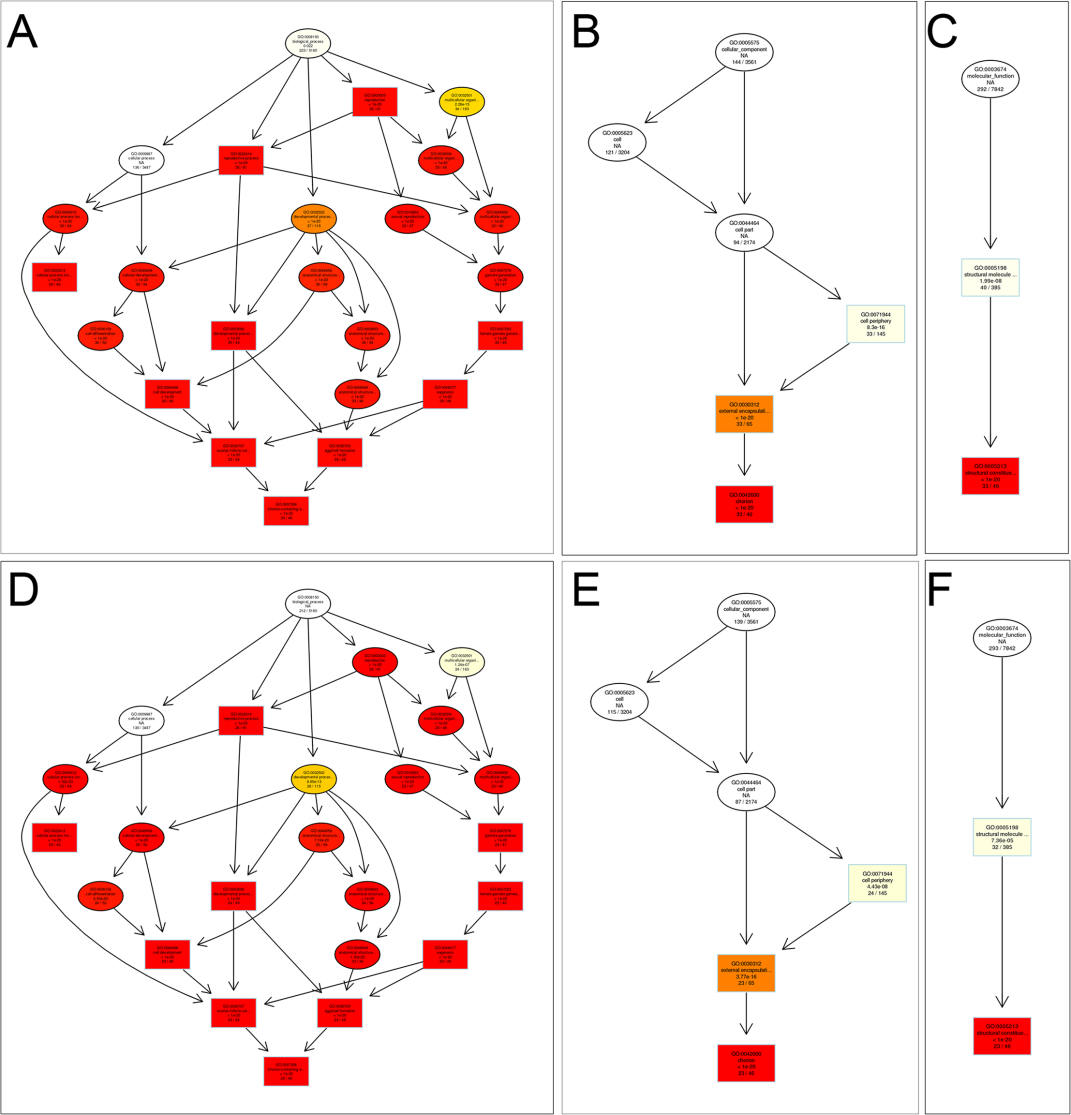
GO enrichment analysis for DEGs between two lines. (A) Biological process for DEGs before thermal induction. (B) Cellular component for DEGs before thermal induction. (C) Molecular function for DEGs before thermal induction. (D) Biological process for DEGs after thermal induction. (E) Cellular component for DEGs after thermal induction. (F) Molecular function for DEGs after thermal induction. The sizes of the circles are proportional to the number of genes associated with the GO term. The arrows represent the relationship between parent-child terms. The color scale indicates the corrected P-value of the enrichment analysis.

These results showed that in non-induced eggs, structural molecule activity and biological process were significantly enriched for 96 upregulated genes in PL. In particular, biological processes included 94 upregulated genes, while 132 downregulated genes in PL were significantly enriched in 33 pathways (S5 Table). As shown in Fig 4A, four main functional categories of reproductive process, reproduction, multicellular organismal process and structural molecule activity were significantly enriched for DEGs between the two lines. More specific terms for these enriched categories were the structural constituent of chorion, chorion-containing eggshell formation and chorion (Fig 5A–5C).

In thermally induced eggs, only structural molecule activity was significantly enriched for three upregulated genes in PL compared with AL, while 31 downregulated genes were significantly enriched in 33 pathways (S6 Table). The major groups of downregulated genes in PL belong to developmental processes involved in reproduction, reproductive processes and the structural constituent of chorion. Four main functional categories of reproduction, reproductive process, multicellular organismal process and structural molecule activity were significantly enriched for DEGs between the two lines (Fig 4B). More specific terms for these enriched categories were structural constituent of chorion, chorion-containing eggshell formation and chorion (Fig 5D–5F).

The results of GO enrichment analysis indicated some differences in the multiple biological processes between the two lines.

### KEGG pathway enrichment analysis of regulated genes

Analysis of DEGs through KEGG [22] showed that 538 DEGs between the two lines in non-induced eggs could be assigned to 72 pathways, while 545 DEGs between two lines in induced eggs were assigned to 74 pathways. There was no enriched pathway observed for DEGs between the two lines before and after thermal induction. The results of KEGG pathway analysis in non-induced and induced eggs are shown in S7 Table and S8 Table, respectively. Metabolic pathways are the major pathways in non-induced and induced eggs, in which 4.1% and 4.7% of associated genes were differentially expressed between the two lines, respectively. Other metabolic pathways were differentially regulated between the two lines, for example carbohydrate metabolism, lipid metabolism, nucleotide metabolism, amino acid metabolism, metabolism of other amino acids, glycan biosynthesis and metabolism, metabolism of cofactors and vitamins, and xenobiotics biodegradation and metabolism. This result indicated that there were some differences in basal metabolism between the two lines.

Many pathways involved in signal transduction, such as the hedgehog signaling pathway, Wnt signaling pathway, Notch signaling pathway, Hippo signaling pathway-fly, Jak-STAT signaling pathway and MAPK signaling pathway-fly, were represented by DEGs between the two lines. Furthermore, the pathways associated with signaling molecules and interaction, such as neuroactive ligand-receptor interaction and ECM-receptor interaction, were represented by a certain number of DEGs. These findings indicated that there are some differences between the two lines’ pathways of signal transduction and signaling molecules and interaction.

Certain DEGs participate in pathways associated with transport and catabolism, such as peroxisome, endocytosis, lysosome phagosome and regulation of autophagy. These findings indicated that changes in transport and catabolism pathways might be related to regulation of parthenogenesis.

The results of pathway enrichment analysis indicated significant differences in the pathways used between the two lines.

## Discussion

Our results demonstrated that PL and AL silkworm eggs have different gene expression patterns. Previously, few works were devoted to the analysis of the transcriptome difference between parthenogenetic and fertilized individuals. An early study by Hanson et al. showed that a large number of genes are involved in the parthenogenetic progress in different species. In insects, a RNA-seq study on the obligate parthenogenetic (OP) and cyclical parthenogenetic (CP) strains of a monogonont rotifer indicated that in these two strains, the expressions of 88% genes overlapped, and several genes that showed increased expression in CP strains were mainly involved in steroid signaling, meiosis, gametogenesis and dormancy, and some genes relating to asexual egg production were highly expressed in OP strains [23]. Microarray analysis on the parthenotes and fertilized embryos developed *in vitro* indicated transcript differences for 749 mouse genes (using a cut-off of 1.8-fold-change). Transcriptomic profile analysis in rabbits indicated that 2541 genes were differentially expressed between parthenotes and normally *in vivo* fertilized blastocysts. In addition, among those DEGs, 76 genes related to DNA and RNA binding were upregulated and 16 genes related to transport and protein metabolic process are downregulated in *in vivo* cultured parthenote blastocysts (using a cut-off of 3-fold-change) [24].

The type and mechanism of parthenogenesis vary between organisms [4], [25]. Therefore, in the present study, we investigated the transcript level of all genes in eggs using RNA-seq to further address the molecular mechanism of silkworm thermal parthenogenesis. To the best of our knowledge, this is the first report of a high-resolution snapshot of the transcriptomic differences between PL and AL in silkworms. In addition, the reliability and accuracy of transcriptional data were validated by qRT-PCR. In this dataset, the numbers of DEGs between PL and AL in non-induced and thermally induced eggs were 538 and 543, respectively. Among the DEGs in the non-induced eggs, there were fewer upregulated genes than downregulated ones, while there were more upregulated genes than downregulated genes in thermally induced eggs. KEGG analysis showed that the DEGs were involved in many crucial processes and pathways, such as metabolic pathways; nicotinate and nicotinamide metabolism; valine, leucine and isoleucine degradation; glutathione metabolism; and pyruvate metabolism. These findings are important for further studies of silkworm thermal parthenogenesis.

In the carbohydrate metabolism pathway, PL contained more downregulated genes than the AL. Among the DEGs in this pathway, some showed large fold-changes, including *putative hydroxypyruvate isomerase* (*DANRE*), *phosphoenolpyruvate carboxykinase* (*PCKG*), *NADP-dependent malic enzyme* (*PHAVU*) and *Ribokinase* (*RBSK*). PCKG is a key enzyme in gluconeogenesis, which is an important metabolic pathway [26]. The main function of gluconeogenesis is to supply glucose as the major fuel to tissues for metabolism [27]. Downregulation of *PCKG* suggested that gluconeogenesis between two lines was different. Two *PHAVU* genes were downregulated in PL and one of them showed the expression difference only after thermal induction (by more than 20-fold). The PHAVU enzyme is widely distributed and is implicated in diverse metabolic pathways [28]. The activity of PHAVU increased in response to certain stresses, including high temperature; this increase was related to the heat shock response to high temperature [29]. Thus, the difference in expression of the *PHAVU* gene after thermal induction may be ascribed to a thermal stability difference between two lines. A larger heat shock response was induced in AL after thermal induction, resulting in changes in the expressions of some heat shock-regulated genes.

In non-induced eggs, *RBSK* was expressed in higher in PL than in AL; however, there was no difference in expression between two lines in the thermally induced eggs. Ribokinase (encoded by *RBSK*) is a member of the superfamily of carbohydrate kinases and participates in the first step of ribose metabolism [30]. D-ribose-5-phosphate is a product of this ribose metabolism, which may subsequently enter the pentose phosphate pathway and is used in the synthesis of amino acids (histidine and tryptophan) [30], [31]. High expression of *RBSK* may be related to the high demand for pentoses in cells of non-induced eggs of PL.

Most DEGs associated with amino acidic metabolism and metabolism of other amino acids pathway were increased in PL, such as *isovaleryl-CoA dehydrogenase*, *mitochondrial* (*IVD*), *ornithine decarboxylase 1* (*DCOR1*), *omega-crystallin* (*CROM*), *5-oxoprolinase* (*OPLA*) and *cysteine synthase* (*CYSK*). In addition, *homocysteine S-methyltransferase 1* (*HMT1*), *kynurenine 3-monooxygenase* (*KMO*), *dihydropyrimidine dehydrogenase* (*DPYD*) and *glycine N-methyltransferase* (*GNMT*) were downregulated in PL. Cysteine synthase, encoded by *CYSK*, is a key enzyme that catalyzes the formation of cysteine from O-acetylserine [32]. It plays an important role in early development of silkworm embryos because of its participation in the degradation of ovovitellin during embryonic development [33]. In silkworms, tryptophan metabolism pathways, which include the products of *KMO* and *CROM*, are involved in the color formation and ommochrome composition of eggs [34]. Changes in the tryptophan metabolism pathway revealed in this study may be associated with the pigmentation difference between the two lines. In the arginine metabolism pathway, ornithine decarboxylase 1 (*DCOR1*) participates in the conversion of arginine to ornithine. Ornithine is further converted into the α-amino acid, which is important for embryonic development through transamination [35].

Of particular interest is the finding that in the translation pathway of PL, there were more upregulated genes than downregulated genes. Genes with high fold-changes, such as *60S ribosomal protein L29* (*RL29*), *polycomb protein l (1) G0020 (U202)* and *aladin (AAA*S), are involved in translation. For example, *RL29*, *RL37* and *RT17* were differentially expressed between two lines. Differential expression of ribosomal protein genes between PLs and ALs in the present work agrees with a previous observation by He et al., who showed that ribosomal protein L7 was differentially expressed between sexual and parthenogenetic reproduction of silkworm eggs [36]. Other work by Hanson et al. [23] also revealed that some *RPL* and *RPS* genes were differentially expressed between OP and CP strains of the monogonont rotifer. Gene *U202* encodes a polycomb protein l (1) G0020 (U202), which plays an important role in remodeling chromatin structure during which epigenetic silencing of genes takes place [37]. The polycomb genes are considered to be the homeotic switch gene regulators that maintain homeotic gene repression through a possible chromatin regulatory mechanism [38]. Miri et al. found that in non-induced parthenogenetic trophoblast stem cells (TSCs), loss of a polycomb gene (*SFMBT2*) resulted in defects in the maintenance of trophoblast cell types necessary for development of the extra-embryonic tissues, particularly the placenta [39]. Therefore, upregulation of *U202* might be important for the success of PL parthenogenesis.

In transport and catabolism pathways, 11 genes were differentially expressed between the two lines in non-induced eggs and 12 differentially expressed in thermally induced eggs. The majority of these DEGs were upregulated in PL, accounting for almost 65% and 75%, respectively, in the non-induced and thermally induced eggs. Genes with high fold-changes, such as *putative fatty acyl-CoA reductase CG5065* (*A1ZAI5*), *transcriptional enhancer factor TEF-3* (*TEAD4*), *polypeptide N-acetylgalactosaminyltransferase 1* (*ACT*), *heat shock protein 68* (*HSP68*) and *mpv17-like protein 2* (*M17L2*), are involved in this pathway. As a mitochondrial inner membrane protein, MPV proteins, encoded by *MPV* genes, are implicated in the metabolism of reactive oxygen species (ROS) [40]. ROS are formed as a natural byproduct of the normal metabolism of oxygen; however, ROS levels are increased dramatically by environmental stresses, such as UV and heat exposure, resulting in significant damage to cellular structures [41], [42]. In the present study, the *MPV-17* and *M17L2* genes were highly expressed in PL, and may be involved in scavenging ROS resulting from thermal induction. *HSP68* was highly expressed in AL and its expression level increased after thermal activation. This gene encodes a 68-kDa heat shock protein, a member of the heat shock protein 70 (HSP 70) family [43]. HSP 70 is activated by heat shock, as well as a wide range of stresses, such as treatment of amino acid analogs, heavy metals and inhibitors of oxidative phosphorylation [44]. High expression of *HSP68* in AL before thermal induction might be associated with stress in response to the dissection of the moth body and washing of the eggs. The increased expression after thermal induction might be related to the stress response to the thermal activation. AL might be unable to adapt to the dissection, washing and thermal activation during this first thermal induction.

In replication and repair pathway, many genes related to DNA repair, such as *DNA repair protein RAD51 homolog 3* (*RA51C*), *DNA repair protein REV1* (*REV1*), *mismatch repair endonuclease PMS2* (*PMS2*) and *WD repeat-containing protein 48 homolog* (*WDR48*), were differentially expressed. In addition to endogenous DNA damage in organisms, DNA damage can also be induced by various environmental stresses and chemicals agents, such as ionizing radiation, UV light and thermal shock [45], [46]. Organisms have evolved several systems to detect DNA damage, signal its presence and mediate its repair [47]. RA51C is a member of the RAD51 protein family, which assists in repairing DNA double strand breaks [48]. REV1 recruits DNA polymerases involved in the translation synthesis (TLS) of damaged DNA [49]. Expression changes of genes involved in the replication and repair pathways suggested that thermal induction caused different levels of DNA damage in the two lines, which led to a regulatory change in gene expression. The more effective DNA repair system of PL could be an important factor for the success of PL thermal parthenogenesis. Increased numbers of DEGs associated with replication and repair pathways emerged after thermal induction. Thus, long-term thermal induction might cause DNA damage in the two lines.

Among the DEGs between the two lines, there were many homologous genes of *CHB1* whose expressions were downregulated in PL. For example, the numbers of *CHBI* genes in non-induced and induced eggs were 38 and 25, respectively. These homologous genes belong to the chorion gene family and were all downregulated in PL, by up to 64-fold at the transcript level. The chorion genes of *B*. *mori* comprise a large multigene family that is expressed in a developmentally complex manner during eggshell formation [50], [51]. In silkworms, chorion complexes are a group of structural protein genes comprising more than 200 members distributed in the early, medium and later stages of oogenesis, with one α- and two β-branches [52]. Many chorion genes are linked, forming at least three clusters on chromosome 2 [53]. The highly expressed chorion genes in AL and their decreased expression after thermal induction indicated that the oogenesis progress to maturity is different between PL and AL.

After thermal induction, most eggs of PL were similar to fertilized eggs; however, only a fraction of the eggs of AL were induced successfully and most of these induced eggs of AL could not hatch offspring. After thermal induction, 125 DEGs were identified that represented new differences between the two lines.

Pigmentation of eggs (silkworm eggs should shift from yellow to brown or gray, even very dark) is the mark of successful thermal induction of silkworm parthenogenesis. The expressions of certain transport-related genes were increased in PL after thermal induction, such as *ATP-binding cassette sub-family G member 4* (*ABCG4*) and *Major facilitator superfamily domain-containing protein 8* (*MFSD8*). ATP-binding cassette sub-family G member 4 (ABCG4) belongs to the ATP-binding cassette (ABC) transporter family, which plays an important role in various biological reactions in all living organisms [54]. In insects, ABC transporters participate in uric acid metabolism, development and, possibly, insecticide resistance [55]. Some ABC members are also involved in the pigment transport progress [56]. Major facilitator superfamily domain-containing protein 8 (MFSD8) is a member of the major facilitator super family (MFS), which is one of the largest groups of secondary active transporters and are conserved from bacteria to humans [57]. MFS proteins play important roles in the pigmentation process [58]. Therefore, upregulation of *ABCG4* and *MFSD8* in PL after thermal induction might be related to enhanced pigment transport, because more eggs were induced successfully in PL, requiring more pigment production and transport.

In addition to the above-mentioned DEGs involved in DNA repair pathways, a DEG emerged after thermal induction that encoded a protein possibly related to DNA repair. *Three prime repair exonuclease 2* (*TREX2*) was upregulated in PL after thermal induction. TREX2 was reported to participate in double-stranded DNA break repair [59]. Alfonso et al. found that a DNA repair protein gene was downregulated in rabbit parthenogenetic blastocysts developed under *in vivo* conditions [24]. We hypothesized that downregulation of DNA repair proteins may be the major reason why parthenogenesis in rabbits can be induced but cannot develop completely. Therefore, high expression of some repair-related genes, including *TREX2*, in silkworm PL after thermal induction may be an important reason for the high parthenogenetic ability of PL.

*RIF1* encodes a telomere-associated protein RIF1, which is involved in capping chromosome ends (telomeres) [60]. RIF1 acts as a negative regulator of telomere length [61]. Yu et al. found that pES cells generated from parthenogenetically activated oocytes exhibit telomere elongation or even slightly longer telomeres compared with fES cells [62]. TAR1, encoded by *TAR1*, is probably involved in auxin production and is required for proper embryo patterning. *TAR1* expression increased in thermally induced eggs of AL, indicating that it might be required for proper embryo patterning, because abnormal embryo development and patterning emerged after thermal shock of AL [63]. TAR1 is closely associated with embryonic development, cell differentiation and oncogenesis.

Interestingly, three *zinc finger protein genes* (*ZFP* genes) were upregulated in AL: *zinc finger protein 57* (*ZNF57*), *zinc finger protein 26* (*ZFP26*) and *zinc finger protein ZPR1* (*ZPR1*). Zinc finger proteins (ZFPs) are a super family of proteins involved in numerous activities during organisms’ growth and development [64]. ZFPs also regulate resistance mechanism to various biotic and abiotic stresses [65]. ZFPs play a role in post-transcriptional regulation of the heat shock response [66]. The upregulation of the three ZFPs in AL after thermal induction might be related to the transcriptional regulation of the heat shock response.

## Conclusions

In conclusion, the present work revealed differences in the parthenogenetic ability between PL and AL of silkworms at the transcript level. Transcriptomic analysis identified many DEGs encoding proteins that are key component for crucial biological processes and signaling pathways, such as carbohydrate metabolism, amino acid metabolism, translation transport and catabolism. These findings provide clues for further investigation of the molecular mechanisms of silkworm parthenogenesis.

## Supporting Information

S1 Table Primers used in quantitative real-time reverse transcription-PCR. (DOC)

S2 Table Statistics of DEGs in non-thermally induced eggs. (XLS)

S3 Table Statistics of DEGs in thermally induced eggs. (XLS)

S4 Table Statistics of DEGs. (XLS)

S5 Table Results of GO enrichment analysis in non-thermally induced eggs. (XLS)

S6 Table Results of GO enrichment analysis in thermally induced eggs. (XLS)

S7 Table Results of KEGG pathway analysis in non-thermally induced eggs. (XLS)

S8 Table Results of KEGG pathway analysis in thermally induced eggs. (XLS)
