# Supplementary material for: Transcriptome Analysis of Thermal Parthenogenesis of the Domesticated Silkworm
Source: PLoS One. 2015 Aug 14;10(8):e0135215. doi: 10.1371/journal.pone.0135215 (PMC4537240; doi:10.1371/journal.pone.0135215)
Supplement: S1 Table — (DOC) [file pone.0135215.s001.doc]

Table S4 Primers used in quantitative real-time reverse transcription-PCR

| Gene name | Sequence 5’-3’ | Primer type |
| --- | --- | --- |
| *60S ribosomal protein L29* | CGAGGACTGGGATGAAGAGA | F |
|  | AAGTGCCCTTGTAATCTGGGTT | R |
| *Heat shock protein 68* | CAATGAAGTGGATGCTGATGGT | F |
|  | ccttgtcaaagactcggaatgc | R |
| *Protein Mpv17* | CACCCTCAAACCACTCTCTGAA | F |
|  | ATGTGCCCATCTTCTCCGA | R |
| *Cysteine synthase* | TCCTGGATGTTGACTGCCTT | F |
|  | GGCAGTCCTCTTATTGGTTG | R |
| *Ribokinase* | CAGAGGCGGAGGAGAACAAA | F |
|  | CACCACTGATACCCTGAAACCT | R |
| *Purine nucleoside phosphorylase* | GATGCTGACCCATACCTCAAGT | F |
|  | AGGGCGGATTGGAAATGA | R |
| *Chorion class B protein L11* | CCTGGATGAAGATGGACGGA | F |
|  | GCTTTGTCCCGTGACTGTGT | R |
| *18s rRNA* | CAGATGGCACTTACACCCGT | F |
|  | TGGAAGAAAGGGTGAGCCA | R |
